# Simple Wound Closure for Civilian Cranial Gunshot Wounds: A Systematic Literature Review

**DOI:** 10.7759/cureus.25187

**Published:** 2022-05-21

**Authors:** Evan M Krueger, Joshua Moll, Rahul Kumar, Victor M Lu, Ronald Benveniste, Joacir G Cordeiro, Jonathan Jagid

**Affiliations:** 1 Neurosurgery, University of Miami Miller School of Medicine, Jackson Memorial Hospital, Miami, USA; 2 Neurosurgery, University of Miami Miller School of Medicine, Miami, USA; 3 Neurosurgery, University of Miami, Miami, USA

**Keywords:** penetrating traumatic brain injury, civilian, cranial, simple wound closure, gunshot wound

## Abstract

Civilian cranial gunshot wounds are common injuries associated with significant morbidity and mortality. Simple wound closure has been previously proposed as an alternative treatment option for a small subset of patients, but the exact outcomes of this strategy are not well-defined. The objective of this paper was to describe the scientific literature reporting simple wound closure of civilian cranial gunshot wounds, its effect on short-term and long-term neurologic outcomes, and rates of seizures and infections. A systematic literature review was performed in accordance with the Preferred Reporting Items for Systematic Reviews and Meta-analyses (PRISMA) guidelines. The strength of evidence was assessed using the Grading of Recommendation, Assessment, Development, and Evaluation (GRADE) criteria. Seventeen studies were found that met inclusion criteria. There was very low strength of evidence that patients treated with simple wound closure can achieve good short and long-term neurologic outcomes. There was very low strength of evidence that simple wound closure has a higher incidence of mortality compared to operative intervention, especially in patients with initial low Glasgow Coma Scale (GCS) scores. There was very low strength of evidence that patients treated with simple wound closure have a small risk of subsequently developing infections or seizures. In conclusion, under most circumstances, neurosurgical operative intervention should be viewed as the optimal treatment for salvageable civilian cranial gunshot wound patients. However, our literature review showed that simple wound closure is safe and viable. More data are needed to determine the appropriate clinical scenario for using this alternative option.

## Introduction and background

The Center for Disease Control and Prevention (CDC) estimated 12.1 per 100,000 firearm-related deaths in 2019 in the United States [1]. Defining optimal management of civilian cranial gunshot wounds is difficult due to multiple confounders but typically involves some type of operative neurosurgical intervention in salvageable patients. Historically, operative management of penetrating brain injuries has trended towards less aggressive surgical debridement [2]. In 2001, the Surgical Management of Penetrating Brain Injury guidelines were published [2]. These state, “treatment of small entrance bullet wounds to the head with local wound care and closure whose scalp is not devitalized and have no ‘significant’ intracranial pathologic findings is recommended” [2].

Given the high incidence of firearm-related penetrating brain injury, there is a need for a contemporary report to further define the appropriate clinical scenario for utilizing simple wound closure for civilian cranial gunshot wounds. We sought to better understand if simple wound closure is safe and efficacious, and what types of carefully selected patients may be appropriate for this treatment strategy. The purpose of this study is to describe the scientific literature reporting simple wound closure of civilian cranial gunshot wounds, its effect on short-term and long-term neurologic outcomes, and rates of seizures and infections.

## Review

Methods

Search Strategy

A systematic literature review was performed in accordance with the Preferred Reporting Items for Systematic Reviews and Meta-analyses (PRISMA) statement [3]. The data and manuscript were formatted in accordance with Meta-analysis Of Observational Studies in Epidemiology (MOOSE) group recommendations [4]. The following clinical questions were asked to identify evidence-based recommendations for simple wound closure of civilian cranial gunshot wounds:

Q1. What is the incidence of good short and long-term neurologic outcomes for patients treated with simple wound closure?

Q2. What is the incidence of mortality for patients treated with simple wound closure?

Q3. What is the incidence of infection for patients treated with simple wound closure?

Q4. What is the incidence of seizures for patients treated with simple wound closure?

A comprehensive literature search of the PubMed, OVID, EMBASE, SCOPUS, and Cochrane Library databases was conducted from their date of establishment through August 23, 2021. Any type of study design that examined simple wound closure of civilian cranial gunshot wounds was queried. The detailed search protocols for the individual databases are provided in Appendix 1.

Study Selection

A set of a priori inclusion and exclusion criteria were developed. Eligible studies must have been published in the English language, have available full-text reports, n ≥3, and have primary descriptive data on human patients treated with simple wound closure. The patient population must have been civilian, age ≥18, and sustained a cranial gunshot wound with suspected or confirmed dural penetration. Excluded were abstracts, unpublished data, military studies, forensic studies, and patients sustaining non-firearm penetrating injuries (i.e. stabbing, shrapnel, debris, buckshot, BB-gun, nail-gun, etc.).

During initial identification, two investigators with medical backgrounds (JM and RK) independently evaluated search results articles’ titles and abstracts for eligibility. Then in screening, two investigators (JM and RK) independently reviewed the full-text articles for inclusion. Any discrepancies in either initial identification or screening were resolved by a third investigator with expertise in this field (EMK). Studies meeting final inclusion then had their bibliographies cross-reference-checked to identify any additional studies meeting inclusion criteria. The systematic literature search and management of full-text articles was done utilizing Covidence software (Covidence systematic review software, Veritas Health Innovation, Melbourne, Australia, available at www.covidence.org).

Data were then extracted from all the included studies’ primary text. Included data points were a subjective or objective description of neurologic outcomes, such as the Glasgow Outcome Scale (GOS), and the incidence of mortality, infection, seizures, and unplanned neurosurgical operation. A good neurologic outcome was defined as GOS ≥4. The National Institutes of Health (NIH) Quality Assessment Tool for Case Series Studies was used to evaluate the internal validity (risk of bias) for individual included studies [5]. The overall strength of evidence for each clinical question was assessed by the Grading of Recommendation, Assessment, Development, and Evaluation (GRADE) criteria [6-7].

Results

Summary of Studies

**Figure 1:**
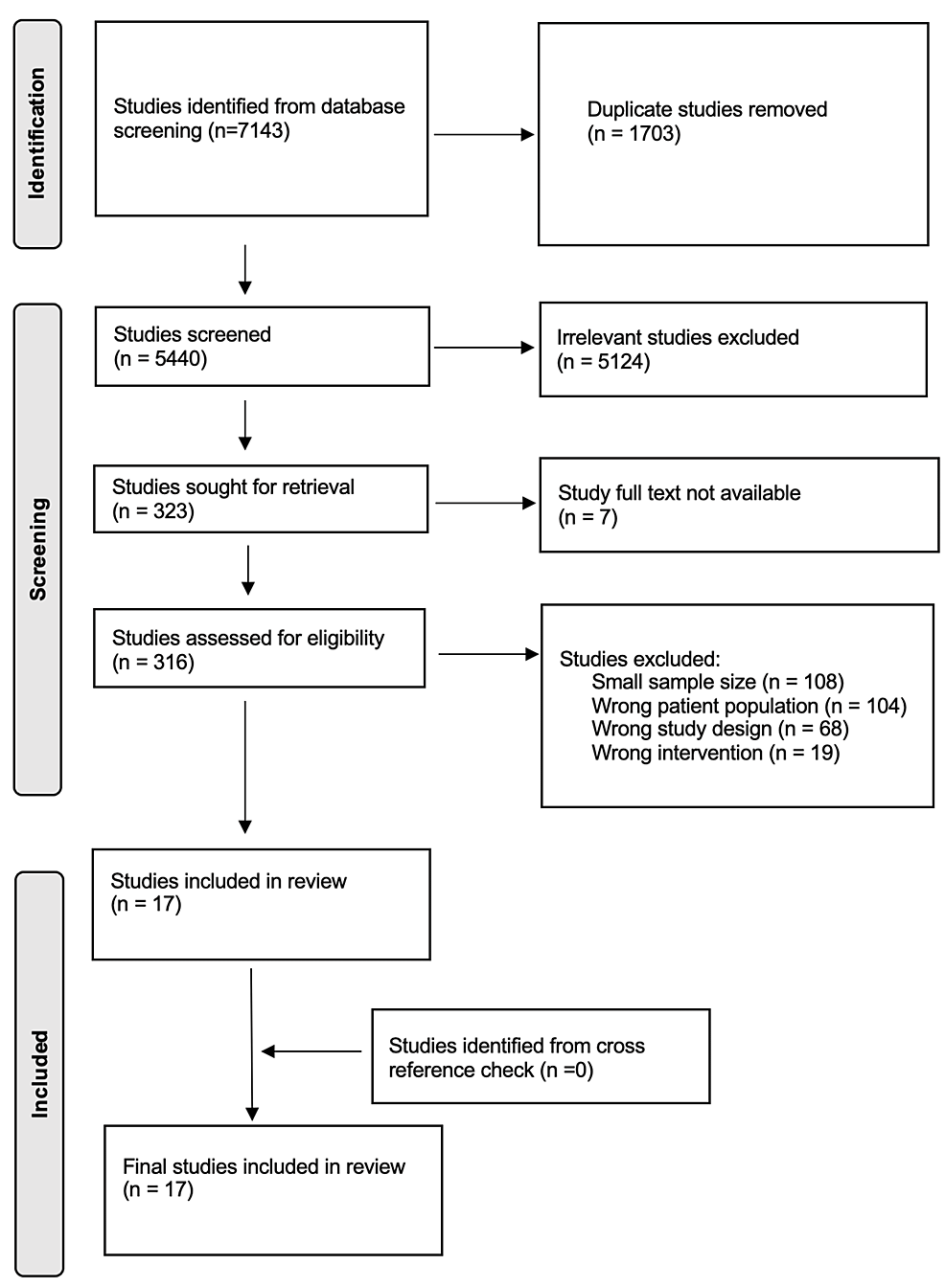
PRISMA Flowchart of Simple Wound Closure for Civilian Cranial Gunshot Wounds PRISMA, Preferred Reporting Items for Systematic Reviews and Meta-Analyses

The literature search yielded 7143 citations, with 316 undergoing full-text review. Ultimately, 17 articles met the final inclusion criteria (Figure 1).

Table 1 summarizes the included studies.

**Table 1:** Summary of Included Studies GCS, Glasgow Coma Scale; ISS; Injury Severity Score; CT, Computed Tomography

Author (year)	Study Duration	Continent	Study Design	N	Criteria	Population
Aarabi et al., (2014) [8]	2000-2002	North America	Multi-center, retrospective chart review	9	•Included: age >16 •Excluded: cause of death not related to a cranial gunshot wound	
D'Agostino et al., (2021) [9]	2006-2016	North America	Multi-center, retrospective chart review	382	•Included: age >17 •Excluded: death within 72 hours	•GCS 3-5: n=158, mean ISS=28.94 •GCS >5: n=224, mean ISS=18.95
De Souza et al., (2013) [10]	1991-2005	South America	Single-center, retrospective chart review	90		•GCS 3-5: n=55 •GCS 6-8: n=8 •GCS 9-12: n=6 •GCS 13-15: n=21
Frosen et al., (2019) [11]	2000-2012	Europe	Single-center, retrospective chart review	40		•median GCS=3
Gressot et al., (2014) [12]	1990-2018	North America	Single-center, retrospective chart review	39	•Included: dural penetration, deemed stable •Excluded: brain death on presentation, died in the emergency room	•mean GCS=4.9
Helling et al., (1992) [13]	1987-1989	North America	Single-center, retrospective chart review	46	•Excluded: death prior to CT scan	•GCS 3-4: n=37 •GCS >4: n=19
Hubschmann et al., (1979) [14]	1973-1975	North America	Single-center, retrospective chart review	37	•Excluded: died in the emergency room, major systemic injuries	
Khan et al., (2014) [15]	1998-2011	Asia	Single-center, retrospective chart review	6	•Excluded: dead on arrival, other non-cranial gunshot wounds	
Kim et al., (2020) [16]	2003-2018	North America	Single-center, retrospective chart review	15	•Included: dural penetration, deemed stable •Excluded: brain death prior to imaging	•mean GCS=12.9 •bilateral reactive pupils: n=12
Kong et al., (2018) [17]	2010-2014	Africa	Single-center, retrospective chart review	71	•Included: single, isolated cranial gunshot wound	
Levy (1999) [18]	1985-1992	North America	Single-center, retrospective chart review, and prospective	86	•Included: GCS>5, isolated cranial gunshot wound •Excluded: intractable hypotension, major systemic injuries	•GCS 6-8: n=5 •GCS 9-11: n=10 •GCS 12-15: n=71
Levy et al., (1994) [19]		North America	Single-center, retrospective chart review, and prospective	130	•Included: GCS3-5 •Excluded: intractable hypotension, major systemic injuries	
Liebenberg et al., (2005) [20]	1996-2003	Europe	Single-center, retrospective chart review	98	•Excluded: dead on arrival, other non-cranial gunshot wounds	
Nagib et al., (1986) [21]	1978-1983	North America	Multi-center, retrospective chart review	20	•Excluded: major systemic injuries, other non-cranial gunshot wounds	•Unilateral multiple lobe injury, or bilateral hemispheric injury: n=20
Petridis et al., (2011) [22]	1993-2008	Europe	Single-center, retrospective chart review	12	•Excluded: other life-threatening injuries	•GCS 3-8: n=9 •GCS 9-15: n=3 •bilateral non-reactive pupils: n=8 •unilateral non-reactive pupils: n=2 •bilateral reactive pupils: n=2
Pikus et al., (1995) [23]	1985-1994	North America	Single-center, retrospective chart review	28		
Raimondi et al., (1970) [24]	1964-1968	North America	Single-center, retrospective chart review	11		

We identified one study that clearly defined the clinicians’ indications for simple wound closure. Kim et al. specifically identified n=15 patients deemed “stable” by the treating physician, were not “futile,” and it was elected for the patient to not receive surgery [16]. As compared to the surgical group, those receiving simple wound closure had significantly fewer bi-hemispheric lesions (20% versus 47.1%; p=.002) and cerebral herniation (6.7% versus 29.4%; p<.0001) [16]. Alternatively, most (16/17, 94.1%) of the included studies did not specify the clinicians’ indications for simple wound closure, i.e. if they were withholding definitive neurosurgical operative intervention and performing simple wound closure instead or if simple wound closure was performed as the perceived ideal definitive treatment.

Summary of Evidence

The overall methodologic quality for all included studies ranged from poor to fair (Table 2).

**Table 2:** Internal Validity (Risk of Bias) Assessment Using the National Institutes of Health (NIH) Quality Assessment Tool for Case Series Studies CD, Cannot Be Determined; NA, Not Applicable

Author (Year)	1. Was the study question or objective clearly stated?	2. Was the study population clearly and fully described, including a case definition?	3. Were the cases consecutive?	4. Were the subjects comparable?	5. Was the intervention clearly described?	6. Were the outcome measures clearly defined, valid, reliable, and implemented consistently across all study participants?	7. Was the length of follow-up adequate?	8. Were the statistical methods well-described?	9. Were the results well-described?	Quality Rating
Aarabi et al., (2014) [8]	Yes	Yes	CD	Yes	No	Yes	Yes	Yes	Yes	Poor
D'Agostino et al., (2021) [9]	Yes	Yes	CD	CD	No	Yes	Yes	Yes	Yes	Poor
De Souza et al., (2013) [10]	Yes	Yes	CD	CD	No	Yes	Yes	Yes	Yes	Poor
Frosen et al., (2019) [11]	Yes	Yes	Yes	Yes	No	Yes	Yes	Yes	Yes	Poor
Gressot et al., (2014) [12]	Yes	Yes	CD	CD	No	Yes	Yes	Yes	Yes	Poor
Helling et al., (1992) [13]	Yes	Yes	CD	Yes	No	Yes	Yes	Yes	Yes	Poor
Hubschmann et al., (1979) [14]	Yes	Yes	CD	CD	No	Yes	Yes	Yes	Yes	Poor
Khan et al., (2014) [15]	Yes	Yes	CD	CD	No	Yes	Yes	Yes	Yes	Poor
Kim et al., (2020) [16]	Yes	Yes	Yes	Yes	Yes	Yes	Yes	Yes	Yes	Fair
Kong et al., (2018) [17]	Yes	Yes	CD	Yes	No	Yes	Yes	Yes	Yes	Poor
Levy (1999) [18]	Yes	Yes	CD	Yes	No	Yes	Yes	Yes	Yes	Poor
Levy et al., (1994) [19]	Yes	Yes	CD	CD	No	Yes	Yes	Yes	Yes	Poor
Liebenberg et al., (2005) [20]	Yes	Yes	CD	Yes	No	Yes	Yes	Yes	Yes	Fair
Nagib et al., (1986) [21]	Yes	Yes	CD	Yes	No	Yes	Yes	Yes	Yes	Poor
Petridis et al., (2011) [22]	Yes	Yes	CD	Yes	No	Yes	Yes	Yes	Yes	Fair
Pikus et al., (1995) [23]	Yes	Yes	CD	CD	No	Yes	Yes	Yes	Yes	Poor
Raimondi et al., (1970) [24]	Yes	Yes	CD	Yes	No	Yes	Yes	Yes	Yes	Poor

The overall strength of evidence for each of our clinical questions was very low (Table 3).

**Table 3:** Strength of Evidence According to the GRADE Methodology ⊕: used to indicate the strength of evidence according to the GRADE methodology with ⊕= very low, ⊕⊕= low, ⊕⊕⊕= moderate, ⊕⊕⊕⊕=high GRADE, Grading of Recommendation, Assessment, Development, and Evaluation

Clinical Question	No. Studies	Baseline Quality	Upgrade	Downgrade	Strength of Evidence
KQ1. Short and Long-Term Outcomes	8	Low	Plausible confounders reducing effect	Limitations in study design Inconsistency of results Indirect comparisons	Very Low ⊕ΟΟΟ
KQ2. Incidence Mortality	14	Low	Plausible confounders reducing effect	Limitations in study design Inconsistency of results Indirect comparisons	Very Low ⊕ΟΟΟ
KQ3. Incidence Infection	3	Low	-	Limitations in study design Indirect comparisons	Very Low ⊕ΟΟΟ
KQ4. Incidence Seizure	2	Very Low	Large magnitude of effect	Limitations in study design Indirect comparisons	Very Low ⊕ΟΟΟ

Q1. What is the incidence of good short and long-term neurologic outcomes for patients treated with simple wound closure?

Three studies described the short-term neurologic outcomes for patients treated with simple wound closure. De Souza et al. reported that 26.6% (24/90) of patients treated with simple wound closure went on to achieve good neurologic outcomes at the time of hospital discharge [10]. The majority of simple wound closure patients (24/26, 92.3%) had initial GCS scores ≥9 [10]. Of all patients with presenting GCS scores ≥9, there were no differences in neurologic outcomes for those treated with simple wound closure versus operative intervention [10]. Second, Petridis et al. reported that 33.3% (4/12) of patients treated with simple wound closure went on to achieve good neurologic outcomes at the time of hospital discharge [22]. Seventy-five percent (3/4) of simple wound closure patients had initial GCS scores ≥9 [22]. Alternatively, 77.8% (7/9) of surgical patients who had initial GCS scores ≥9 went on to achieve good neurologic outcomes; however, no statistical comparison was made between simple wound closure and surgical patients with initial GCS scores ≥9 [22]. Finally, one study simply noted that 27.3% (3/11) of patients treated with simple wound closure had neurologic deficits (hemiparesis and hemianopia) at the time of hospital discharge [24].

Five studies described the long-term neurologic outcomes for patients treated with simple wound closure. Kim et al. reported that stable patients treated with simple wound closure (mean follow-up: 35.6 months) versus surgical patients (mean follow-up: 33.3 months) had a similar incidence of good long-term neurologic outcomes (75% versus 58.3%, p=.17), although simple wound closure patients had higher mean admission GCS scores compared to surgical patients (12.9 versus 7.5; p<.0001) [16]. Conversely, Gressot et al. reported that simple wound closure patients have a lower incidence of good neurologic outcomes at six months compared to surgical patients (3/39, 7.7% versus 20/80, 25%; p=.034, OR 4.01, 95% CI 1.11-14.5) although simple wound closure patients had lower mean admission GCS scores compared to surgical patients (4.9 versus 6.5; p=.012) [12]. Third, Levy reported the incidence of good neurologic outcomes within 12 months of injury for patients treated with simple wound closure was 0% (0/5) for those presenting with an initial GCS 6-8, 50% (5/10) for those presenting with an initial GCS of 9-11, and 95% (67/71) for those with an initial presenting GCS of 12-15 [18]. Next, Levy et al. reported that in patients who presented as GCS 3-5 and were treated with simple wound closure, .8% (1/130) survived and had a GOS score of 2 at the six and 12-month follow-ups [19]. Lastly, Aarabi et al. reported that 22.2% (2/9) of patients treated with simple wound closure achieved good neurologic outcomes at a mean 65-month follow-up [8].

Using the GRADE methodology, there is very low strength of evidence that patients treated with simple wound closure can achieve good short and long-term neurologic outcomes (Table 3).

Q2. What is the incidence of mortality for patients treated with simple wound closure?

A total of 14 studies were found that reported the incidence of mortality in patients treated with simple wound closure. Of these, eight studies statistically compared the incidence of mortality for patients treated with simple wound closure versus patients treated with surgery. Levy et al. reported that in patients with initial GCS 3-5, there was a higher incidence of mortality in patients treated with simple wound closure compared to surgery (129/130, 99.2% versus 37/60, 61.7%; p<.000001) [19]. Similarly, D’Agostino et al. reported that in patients with initial GCS 3-5, simple wound closure patients had a higher incidence of mortality compared to surgical patients (72.2% versus 18.5%; p<.0001) [9]. The same paper also reported no differences in mortality between the two treatment groups for patients with initial GCS ≥6 (8.5% wound closure versus 7.5% surgery; p=.72) [9]. Helling et al. noted a higher incidence of mortality for simple wound closure patients compared to surgery both in patients with initial GCS 3-4 (36/37, 97.3% versus 7/11, 63.6%; p<.0001) and in patients with initial GCS ≥5 (10/19, 52.6% versus 3/16, 18.8%; p=.007) [13]. A statistically significant higher incidence of mortality for patients treated with simple wound closure compared to surgical intervention was also reported by Petridis et al. (8/12, 66.6% versus 6/18, 33.3%; p<.05), Gressot et al. (71.8% versus 37.5%; OR 4.48, CI 1.91-10.5, p=.001), and Frosen et al. (39/40, 98% versus 7/22, 32%; p<.001); however these three studies did not stratify patients by GCS [11-12,22]. Kong et al. reported no differences in mortality for simple wound closure patients compared to surgical patients (16/71, 22.5% versus 6/31, 19.4%, p=.799) [17]. Kim et al. reported that in clinically stable patients, simple wound closure had a lower incidence of mortality compared to surgery (0% versus 16%, p=.01) [16].

Levy stratified the incidence of mortality by GCS for those treated with simple wound closure and reported the incidence of mortality was 100% (5/5) for those presenting with an initial GCS of 6-8, 30% (3/10) for those presenting with an initial GCS of 9-11, and 2% (1/71) for those with an initial presenting GCS of 12-15 [18]. Additionally, five studies simply reported the raw incidence of mortality for patients treated with simple wound closure without any direct statistical comparisons or subcategorizations, and these rates ranged from 33.3% to 100% [8,14-15,20-21,23].

Using the GRADE methodology, there is very low strength of evidence that simple wound closure has a higher incidence of mortality compared to surgical intervention for patients presenting with GCS ≤5. Less data was available that suggested an acceptable risk of mortality for patients with high initial GCS scores treated with simple wound closure.

Q3. What is the incidence of infection for patients treated with simple wound closure?

Three studies reported the incidence of infection for patients treated with simple wound closure. First, D’Agostino et al. clearly defined a central nervous system infection as an empyema, meningitis, ventriculitis, or cerebral abscess [9]. Infection rates were significantly less in the non-operative group compared to the operative group for patients with GCS 3-5 (6/158, 3.9% versus 14/122, 11.5%; p=.016), and for patients with GCS ≥6 (5/224, 2.2% versus 15/214, 7.0%; p=.017) [9]. Second, Kim et al. reported no differences in the incidence of infection for patients treated with simple wound closure compared to operative intervention (0% versus 16.7%; p=.19) [16]. Third, Petridis et al., reported a 16.7% (2/12) incidence of sepsis, 0% (0/12) incidence of meningitis, and 0% (0/12) incidence of cerebral abscess in non-operative patients; as compared to 5.6% (1/18) incidence of sepsis, 0% (0/18) incidence of meningitis, and 0% (0/18) incidence of cerebral abscess in operative patients [22].

Using the GRADE methodology, there is very low strength of evidence that patients treated with simple wound closure have an acceptably low risk of infection (Table 3).

Q4. What is the incidence of seizures for patients treated with simple wound closure?

Two studies described the incidence of seizures in patients treated with simple wound closure. One study reported no differences in the incidence of seizures during hospital admission in patients treated with simple wound closure as compared to surgery (0% versus 8.3%; p=.37) [16]. Similarly, another study reported the incidence of seizures during hospital admission to be 0% [22].

Using the GRADE methodology, there is very low strength of evidence that patients treated with simple wound closure have an acceptably low risk of seizures (Table 3).

Discussion

Q1. what is the incidence of good short and long-term neurologic outcomes for patients treated with simple wound closure?

In terms of short-term neurologic outcomes, two studies showed that 26.6-33.3% of patients with initial GCS ≥9 treated with simple wound closure go on to achieve good neurologic outcomes at the time of hospital discharge [10,22]. However, GCS was subjectively grouped; both studies did not specify the exact length of hospital stay, and one study did not provide a specific p-value other than noting it was not significant when comparing short-term outcomes in simple wound closure versus operative intervention [10,22]. In terms of good long-term neurologic outcomes in patients treated with simple wound closure, the incidence ranged from 0.8% to 75% [8,12,16,19]. This highly variable treatment effect for neurologic outcomes was likely confounded by a multitude of factors, most notably the initial neurologic exam.

Q2. What is the incidence of mortality for patients treated with simple wound closure?

We found a diverse range of mortality incidence for patients treated with simple wound closure, ranging from 2% to 100%; likely due to a radiographic and clinically heterogeneous population. Mortality for simple wound closure patients was consistently reported as higher compared to operative intervention for patients with very low initial GCS scores. However, there was not a clear mortality benefit for operative intervention as compared to simple wound closure in patients with initial high GCS scores.

Q3. What is the incidence of infection for patients treated with simple wound closure?

We found minimal data describing the incidence of infection in patients treated with simple wound closure; although all studies reported lower or comparable incidence of infection in patients treated with simple wound closure compared to surgical intervention. Several studies did not clearly define their definition of infection, whether this specifically only included central nervous system involvement (CNS), or whether hematogenous seeding of the CNS would constitute an infection.

Q4. What is the incidence of seizures for patients treated with simple wound closure?

We found minimal data describing the incidence of seizures in patients treated with simple wound closure. Both studies reported the incidence of seizures as 0% during hospital admission. This extreme value is limited in clinical plausibility, likely due to the bias of a small and retrospective sample size. Additionally, no long-term seizure data was reported. It is unknown if any hypothetical short or long-term seizures would be secondary to the initial injury, or due to residual bone and foreign body fragments that could not be removed with simple wound closure. However, there is evidence that retained bone and foreign body fragments may not place a patient at significant risk for delayed epilepsy [25-28].

Limitations

There are limitations to this study. First, in regards to fundamental issues with our clinical questions, we were often making indirect comparisons with the intervention in question (simple wound closure). Most studies were not clear if the non-operative patients had definitive neurosurgical intervention withheld and simple wound closure offered alternatively or were given a simple wound closure as the perceived best treatment option. To properly evaluate simple wound closure for civilian cranial gunshot wounds (traditionally viewed as an alternative treatment method), a propensity-matched control comparison group of patients undergoing neurosurgical intervention (traditionally viewed as the standard of care) is necessary. Next, in regards to our methodology, our search strategy could have been refined to screen fewer articles. We did not include a date range in our literature, which could have resulted in studies that we performed with different levels of technology, namely, a CT scanner. While there have been several reports of simple wound closure for military cranial gunshot wounds and for compound depressed skull fractures, which are both somewhat analogous to civilian cranial gunshot wounds, we choose to exclude these types of studies due to multiple confounding variables. We did not include non-English articles due to a lack of resources available for translation. Lastly, in regards to the quality of data analyzed, we identified relatively few studies that met the inclusion criteria. These studies were all of a lower quality of evidence and at risk for reporting bias. There was significant heterogeneity in the data itself, as well as the studies’ explanations and interpretations, which limited our ability to perform a true meta-analysis. It is also difficult to generalize data collected from different countries and health care systems with variable resources and care delivery systems.

Future directions

A small subset of viable civilian patients sustaining an intracranial gunshot wound may present without a mass lesion significantly negatively impacting their neurologic status, without high-risk infectious or epileptogenic features, and/or with unacceptable surgical risk. To offer the best chance of optimal neurologic outcomes for these carefully selected patients, simple wound closure may be a viable option. A study detailing the outcomes and clinical course of civilians sustaining cranial gunshot wounds treated with simple wound closure would be helpful to evaluate the efficacy and limitations of this treatment.

## Conclusions

There is very low strength of evidence suggestive that simple wound closure is a safe and viable treatment option in carefully selected patients. However, it remains unclear how this treatment compares to surgical intervention and what types of patients may be appropriate for simple wound closure. Ultimately, more data are needed to determine the appropriate selection criteria for simple wound closure in civilian cranial gunshot wound patients. Based on our shared clinical experience and the currently available literature, most salvageable civilian patients sustaining a cranial gunshot wound should be treated with operative neurosurgical intervention to improve neurologic outcomes and reduce mortality.

## Appendices

Appendix 1: text search strategy

PubMed: 2045 articles

(("wounds, gunshot"[MeSH Terms] OR "penetrat*"[All Fields] OR "pierc*"[All Fields] OR "gsw"[All Fields] OR "ptbi"[All Fields] OR "pbi"[All Fields] OR "pbt"[All Fields])

AND

("skull"[MeSH Terms] OR "head"[MeSH Terms] OR "cerebrum"[MeSH Terms] OR "brain"[MeSH Terms] OR "craniocerebral"[All Fields])

AND

("wounds and injuries"[MeSH Terms] OR "trauma*"[All Fields] OR "injur*"[All Fields]))

AND

((booksdocs[Filter] OR casereports[Filter] OR clinicalstudy[Filter] OR clinicaltrial[Filter] OR clinicaltrialprotocol[Filter] OR clinicaltrialphasei[Filter] OR clinicaltrialphaseii[Filter] OR clinicaltrialphaseiii[Filter] OR clinicaltrialphaseiv[Filter] OR comparativestudy[Filter] OR controlledclinicaltrial[Filter] OR dataset[Filter] OR journalarticle[Filter] OR meta-analysis[Filter] OR multicenterstudy[Filter] OR observationalstudy[Filter] OR pragmaticclinicaltrial[Filter] OR randomizedcontrolledtrial[Filter]) AND (fft[Filter]) AND (humans[Filter]) AND (english[Filter]) AND (adolescent[Filter] OR alladult[Filter] OR adult[Filter] OR middleagedaged[Filter] OR middleaged[Filter] OR aged[Filter] OR 80andover[Filter] OR youngadult[Filter]))

EMBASE: 2673 articles

gunshot wound*/exp

penetrat*:ti,ab,kw

pierc*:ti,ab,kw

gsw:ti,ab,kw

ptbi:ti,ab,kw

pbi:ti,ab,kw

pbt:ti,ab,kw

OR/1-7

skull:ti,ab,kw

head:ti,ab,kw

cerebrum:ti,ab,kw

brain:ti,ab,kw

craniocerebral:ti,ab,kw

cranial:ti,ab,kw

OR/9-14

'wounds and injuries'/exp

trauma*:ti,ab,kw

injur*:ti,ab,kw

OR/16-18

8 AND 15 AND 19

20 AND 'human'/de AND ([adolescent]/lim OR [adult]/lim)

OVID: 787 articles

("gunshot wounds" OR penetrat* OR pierc* OR GSW OR ptbi OR pbi OR pbt) AND (skull OR head OR cerebrum OR brain OR craniocerebral OR cranial) AND ("wounds and injuries" OR trauma* OR injur*)

limit to (english language and full text and humans and ("all adult (19 plus years)" or "adolescent (13 to 18 years)"))

Cochrane Library: 214 articles

("gunshot wounds" OR penetrat* OR pierc* OR GSW OR ptbi OR pbi OR pbt) AND (skull OR head OR cerebrum OR brain OR craniocerebral OR cranial) AND ("wounds and injuries" OR trauma* OR injur*) in All Text - (Word variations have been searched)

Scopus: 1424 articles

TITLE-ABS-KEY ( ( "gunshot wounds"  OR  penetrat*  OR  pierc*  OR  gsw  OR  ptbi  OR  pbi  OR  pbt )  AND  ( skull  OR  head  OR  cerebrum  OR  brain  OR  craniocerebral  OR  cranial )  AND  ( "wounds and injuries"  OR  trauma*  OR  injur* ) )  AND  ( LIMIT-TO ( LANGUAGE ,  "English" ) )  AND  ( LIMIT-TO ( EXACTKEYWORD ,  "Human" )  OR  LIMIT-TO ( EXACTKEYWORD ,  "Humans" ) )  AND  ( EXCLUDE ( EXACTKEYWORD ,  "Child" )  OR  EXCLUDE ( EXACTKEYWORD ,  "Nonhuman" )  OR  EXCLUDE ( EXACTKEYWORD ,  "Child, Preschool" )  OR  EXCLUDE ( EXACTKEYWORD ,  "Traffic Accident" )  OR  EXCLUDE ( EXACTKEYWORD ,  "Animals" )  OR  EXCLUDE ( EXACTKEYWORD ,  "Preschool Child" )  OR  EXCLUDE ( EXACTKEYWORD ,  "Infant" )  OR  EXCLUDE ( EXACTKEYWORD ,  "Thorax Injury" )  OR  EXCLUDE ( EXACTKEYWORD ,  "Drug Penetration" )  OR  EXCLUDE ( EXACTKEYWORD ,  "Abdominal Injury" )  OR  EXCLUDE ( EXACTKEYWORD ,  "Stab Wound" )  OR  EXCLUDE ( EXACTKEYWORD ,  "School Child" )  OR  EXCLUDE ( EXACTKEYWORD ,  "Face Injury" )  OR  EXCLUDE ( EXACTKEYWORD ,  "Animal" )  OR  EXCLUDE ( EXACTKEYWORD ,  "War" )  OR  EXCLUDE ( EXACTKEYWORD ,  "Neck Injury" )  OR  EXCLUDE ( EXACTKEYWORD ,  "Falling" )  OR  EXCLUDE ( EXACTKEYWORD ,  "Unclassified Drug" )  OR  EXCLUDE ( EXACTKEYWORD ,  "Human Tissue" )  OR  EXCLUDE ( EXACTKEYWORD ,  "Orbit" )  OR  EXCLUDE ( EXACTKEYWORD ,  "Eye Injury" )  OR  EXCLUDE ( EXACTKEYWORD ,  "Neck Injuries" )  OR  EXCLUDE ( EXACTKEYWORD ,  "Blast Injury" )  OR  EXCLUDE ( EXACTKEYWORD ,  "Laceration" )  OR  EXCLUDE ( EXACTKEYWORD ,  "Spinal Cord Injury" )  OR  EXCLUDE ( EXACTKEYWORD ,  "Accidents, Traffic" )  OR  EXCLUDE ( EXACTKEYWORD ,  "Stroke" )  OR  EXCLUDE ( EXACTKEYWORD ,  "Battle Injury" ) )  AND  ( EXCLUDE ( DOCTYPE ,  "le" )  OR  EXCLUDE ( DOCTYPE ,  "sh" ) )  AND  ( EXCLUDE ( EXACTKEYWORD ,  "Blunt Trauma" )  OR  EXCLUDE ( EXACTKEYWORD ,  "Pathology" )  OR  EXCLUDE ( EXACTKEYWORD ,  "Nuclear Magnetic Resonance Imaging" )  OR  EXCLUDE ( EXACTKEYWORD ,  "Magnetic Resonance Imaging" )  OR  EXCLUDE ( EXACTKEYWORD ,  "Cause Of Death" )  OR  EXCLUDE ( EXACTKEYWORD ,  "Forensic Pathology" )  OR  EXCLUDE ( EXACTKEYWORD ,  "Headache" )  OR  EXCLUDE ( EXACTKEYWORD ,  "Disease Association" )  OR  EXCLUDE ( EXACTKEYWORD ,  "Physiology" )  OR  EXCLUDE ( EXACTKEYWORD ,  "Forensic Medicine" )  OR  EXCLUDE ( EXACTKEYWORD ,  "Forensic Ballistics" )  OR  EXCLUDE ( EXACTKEYWORD ,  "Blood Transfusion" )  OR  EXCLUDE ( EXACTKEYWORD ,  "Intracranial Aneurysm" )  OR  EXCLUDE ( EXACTKEYWORD ,  "Osteosynthesis" )  OR  EXCLUDE ( EXACTKEYWORD ,  "Cadaver" ) )
